# Tackling Airborne Virus Threats in the Food Industry: A Proactive Approach

**DOI:** 10.3390/ijerph18084335

**Published:** 2021-04-19

**Authors:** Tahl Zimmerman, Shahida Anusha Siddiqui, Werner Bischoff, Salam A. Ibrahim

**Affiliations:** 1 Food Microbiology and Biotechnology Laboratory, Department of Family and Consumer Sciences, College of Agriculture and Environmental Sciences, North Carolina A& T State University, 1601 East Market Street, Greensboro, NC 27411, USA; ibrah001@ncat.edu; 2 Technical University of Munich Campus Straubing for Biotechnology and Sustainability, Essigberg 3, 94315 Straubing, Germany; shahidasiddiqui777@gmail.com; 3 Food Data Group, German Institute of Food Technologies, 49610 D-Quakenbrück, Germany; 4 Infection Prevention and Health System Epidemiology, Wake Forest School of Medicine, Medical Center Boulevard, Winston-Salem, NC 27157, USA; wbischof@wakehealth.edu

**Keywords:** health, meat processing, airborne virus, COVID-19, food industry

## Abstract

The current SARS-COVID-19 crisis has demonstrated the dangers that airborne virus (AV) pandemics pose to the health of all workers (particularly in the meat processing industry), the economic health of the food industry, and food security. The impact that the current pandemic has had on the food industry points to the need for a proactive rather than reactive approach towards preventing future AV outbreaks. Such a proactive approach should be based on empirical assessments of current AV food safety practices and the development of more robust practices tailored to the culture and needs of the food industry. Moreover, a proactive approach is necessary in order to better prepare the food industry for future AV outbreaks, protect the health of workers, reduce disparities in AV occupational health risks, and enhance the safety of the food supply chain. The aim of this review is to make the case for a new food safety research paradigm that incorporates the intensive study of airborne viruses under conditions that simulate food industry work environments.

## 1. Introduction

History has shown us that pandemics are not new, from the Spanish flu to the more recent outbreaks of Ebola and SARS. In the future, pandemics will likely occur with increasing frequency due to, among other factors, globalization and international mobility, population growth, urbanization, weather related issues in addition to current practices in place in food production systems. We must learn from these past and current pandemics in order to be more prepared for future global outbreaks. This has never been as clear as today, with the disastrous effects the current COVID-19 disaster has had on the population and our economy. There are also many lessons to be learned about the effects of this pandemic on front-line workers, including food industry workers. Learning from this pandemic also means learning what sorts of models are needed to carry out the research necessary to create the knowledge we will need to prepare for the future. In the case of the Food Industry a new food safety model is necessary for guarding against airborne illnesses, rather than only food-borne ones.

## 2. An Airborne Virus Model of Food Safety

Between 1 March and 31 May of last year, 8978 workers in 742 food and agriculture workplaces in 30 states in the US had confirmed COVID-19 [1]. Among agriculture workplaces, meat and poultry processing (MPP) plants are particularly vulnerable to COVID-19 outbreaks. As of 21 July, MPP plants were associated with an estimated 236,000 to 310,000 SARS-COVID-19 cases (6% to 8% of total cases in the US) and 4300 to 5200 deaths (3% to 4% of total US deaths). These numbers included cases in worker communities in which MPP plants were located [2]. A global study on SARS-CoV-2 infection clusters found that food processing plants formed the third largest size clusters. The median cluster size from these installations was 70 primary or secondary infections, which were smaller in magnitude only to cruise ships and prisons. Among the twenty outbreak clusters found in food processing plants around the world, 14 occurred in MPP plants (see Table 1). In MPP plants, the median number of cases per cluster was even higher [3]. These outcomes illustrate the effects of the strong centralization in the food and especially the meat industry [4]. MPP plants are particularly rapid SARS-CoV-2 incubators [5].

Importantly, the mere presence of an MPP plant is associated with transmission in the community where the plant is located, indicating that MPP plants themselves may have functioned as vectors of transmission [2]. The vulnerability of MPP workers to SARS-COVID-19 is so well-acknowledged that these workers are second only to health professionals in priority to receive COVID-19 vaccinations [3].

Outbreaks in meat processing plants have also led to plant closures [4], which led to meat shortages in the early months of the epidemic [5]. In addition, the closures and shortages resulted in 13.6 billion in economic losses in the US by April 2020 alone. Shortages are a natural consequence of the highly centralized nature of the food industry and the resultant just-in-time production strategy that eschews stockpiling [4]. This centralization is particularly severe in the US pork industry where the four largest companies in each meat sector control between 55% and 85% of their respective markets. As another example, only 12 plants in the US are responsible for 50% of pork production, and another 12 for beef production [6]. Absenteeism due to illness can also be disruptive as a 25% increase in absenteeism can lead to a 45% decrease in the food supply [7,8]. This also depends upon the techniques that are used to process the complexities. Therefore, the safety of food industry workers is not only critical for maintaining the workers’ health, but for maintaining the economic and distribution health of the entire meat production system. 

The importance of securing the food system was reflected in the executive orders in the IS declaring meat processing plants to be essential facilities, which led to the reopening of plants in the US previously closed as a consequence of outbreaks [9]. However, this was a reactive measure that balanced the health risks to workers and surrounding communities against the need to keep the population fed, rather than a proactive measure designed to protect both. As a result, the trend today is to close plants temporarily for a single day for cleaning and disinfection [10]. The SARS-COVID-19 epidemic has clearly exposed vulnerabilities in the supply chain, suggesting that Airborne Virus (AV) food safety is in fact somewhat synonymous with food security. As a result, it is incumbent upon food safety researchers to establish the empirically derived Airborne Viral Threat (AVT) food safety practices that work best in food industry settings. Such best-practices that include screening (e.g., self-assessments and temperature taking), medical leave rules, and personal hygiene recommendations can then be used as part of an overall strategy for preventing AVT in MPP facilities. These best practices may subsequently serve as a basis for the implementation of AVT prevention in food safety management systems, such as the hazard analysis and critical control point (HACCP) principles. These systems are currently largely focused on preventing transmission of infectious diseases into and via food along the supply chain, rather than AVT transmission between workers. Including measures against AVT transmission in such food safety management systems would enable prevention and control rather than just reactive measures against AV outbreaks [11].

Pre-SARS-COVID-19, meat packing in the US was already the most hazardous of all occupations with a high rate of illnesses and injuries, in addition to being low-paying [12]. Meat processing workers are highly vulnerable on a number of fronts as they are largely non-unionized [12], many are recent immigrants, and of those, many are disproportionately undocumented. These realities make it difficult for these workers to have any recourse with regard to safety issues. Forty-five percent of MPP workers are low income, 44% are Hispanic, 23% are Black, and 52% are immigrants. Importantly, these percentages go up among assembly line workers where the risk of illness also increases [13]. Fourteen percent of assembly line workers are undocumented and therefore do not have access to healthcare and worker protections that support the prevention and treatment of COVID-19 [14]. The above mentioned issues also increase the occurrence of presenteeism, coming to work despite being sick, which further increases infections at the workplace [15]. In summary, it is unsurprising that 80% of all COVID-19 cases in the meat processing industry occur among racial and ethnic minorities [13]. Moreover, employees receive incentives to continue working while sick, which discourages self-isolation and increases the risk of transmitting AVs to fellow workers [14,16]. Therefore, there is a social justice aspect, both in terms of class and ethnicity, to ensuring MPP worker safety in anticipation of future AVTs. This is because highly vulnerable and low-paid MPP workers from ethnic minorities are at higher risk of contracting AVs. Reducing this risk will help reduce the disparity in AV risk faced by different segments of the population.

As several promising vaccines have been developed against SARS-COVID-19, it is our hope and expectation is that the current crisis will eventually pass. Nevertheless, the disastrous outcome of the current crisis has demonstrated the need for proactively preparing for future AVT crises. In addition, much more needs to be explored regarding the role of animals in the ongoing pandemic, and best practices to protect workers at food processing facilities. 

The World Health Organization (WHO) predicts future infectious disease pandemics [17]. Indeed, the frequency of disease outbreaks has increased over the last few decades, largely as a consequence of deforestation, species extinction [18], population growth, pollution, and climate change [18]. Between 2011 and 2018, the WHO has documented 1483 epidemic events in 172 countries (GPMB 2019), and six international health emergencies have been announced since 2009 [15]. Well-known viral outbreaks that have occurred over the last two decades (other than SARS COVID-19), include SARS (2003), MERS-COV (2012 and 2020), H1N1 (2009), and Ebola (2014). In order to prepare for the next crisis that is likely to occur, proactive and industry specific research into AVT is required.

Regarding SARS-COVID-19 safety measures, early in the SARS-COVID-19 pandemic, the Center for Disease Control (CDC) recommended safety standards that included handwashing hygiene, strengthening medical leave practices [15,19], social distancing, sanitizing surfaces, wearing cloth masks, using partitions, minimizing the use of fans, and adding handwashing or hand sanitizer stations [20]. However, there are no CDC guidelines on additional or novel measures that are specific to food processing [21,22]. Any responses to safety in the area of food processing were reactive in nature as it was unknown a priori if the measures in place at the time were sufficient to stem the tide of infections. We now know that the measures that were in place are indeed insufficient because outbreaks are continuing to occur in meatpacking facilities [10]. 

CDC recommendations are optional, and therefore may not be universally applied in different MPP facilities [12]. In addition, there are no CDC guidelines on additional and novel measures that are specific for food processing contexts [23] as current CDC guidelines appear to be drawn from the general knowledge of what is understood about infectious diseases, rather than specific guidelines based on AVT in an MPP context. For example, hand hygiene is the most effective way to prevent the spread of infectious diseases generally, yet the availability of this measure only offers limited protection from viruses such as COVID-19 that are spread by respiratory droplets [24]. Physical barriers have been used for decades in the healthcare and food service industry to prevent the movement of pathogen-carried respiratory droplets [18], but the effectiveness of such barriers on the assembly line, or in the context of airflow in an MPP is less well-known. Importantly, as mentioned above, SARS-COVID-19 does not generally spread via surface contacts, but rather from person to person via respiratory droplets. Therefore, researching the dynamics of AVT transmission through the air should be prioritized in the context of improving food safety protocols designed to protect against AVTs, rather than the current trend in AV food safety research, which focuses on the efficacy of surface disinfectants in decontaminating SARS-COVID-19 from surfaces [25,26]. 

An effective technique against nosocomial viruses is fogging with H_2_O_2_ [27], but it can only be carried out in the absence of employees or food due to its skin- and eye-irritating properties, raising operational and additional food safety concerns [15]. Ozone is a gaseous alternative to inactivate Viruses, especially effective at high relative humidity [28]. However, it poses health risks to humans and is not recommended to be used above concentrations of 0.1 ppm, with no information on long term effects [29,30] and only scarce research on effectiveness against SARS-CoV-2 [30]. Alternatively, UV-light has proven effective against AVs [31]. In the food industry, “in-duct” systems are recommended, where air is irradiated after passage through HVAC systems. These systems allow sanitation of the air without workers coming into contact with radiation [32]. HVAC systems and air exchange in general are recognized as important factors for AVT, especially in relation to SARS-CoV-2. Minimum air exchange rates should be adhered to, and flow of recycled air along stationary persons avoided [31,33].

The topic of SARS-CoV-2 transmission specifically in a food processing context has only been taken up in scientific literature. In order to adhere to physical distancing recommendations, multiple approaches have been proposed. These approaches include staggering workstations and side-by-side workstations meant to prevent workers from facing each other, adopting facemask policies according to national guidelines pacing out workstations, limiting the number of workers in an area at a given time, and organizing workers into teams with little inter-team contact. Also, attention is given to ensuring chemical sanitation by proper accessibility to and use of sanitizers [15].

Meanwhile, new risk factors associated with increased COVID-19 transmission at MPP facilities have been identified (See Table 2). These factors include (1) long work shifts (8–12 h); (2) close and prolonged proximity to other workers (<6 feet; >15 min) [13]; (3) difficulties in maintaining proper face covering due to physical demands; (4) shared work spaces [13]; (5) shared transportation [19]; (6) the size of the facility [26]; (7) higher assembly line speeds [2]; (8) temperatures of 0–12 °C degrees, associated with a higher risk of contracting COVID-19 [27,28]; (9) a fast work pace that may prevent the appropriate donning and doffing of masks [15]; (10) the high relative humidity of an MPP (90–95%), associated with longer distance movement of respiratory particles [29]; (11) and, additicnally, the cooling systems themselves may also spread COVID-19 by carrying bioaerosols over long distances, as can air flow generally. In an outbreak at a German processing plant, transmission distance was thus increased to at least 8 m as a consequence of recirculation [30,34]. 

Processing environments such as MPPs are generally recognized as medium risk by the WHO because no direct contact with known or suspected cases is intended. However, it is recommended to take into account different working conditions and associated risk factors for each individual workplace [35,36]. MPP-specific information and mitigation measures would be therefore being very helpful in this regard.

More importantly, the above factors are derived epidemiologically and remain empirically untested in a food safety setting. New models that simulate the real-life conditions of an MPP must be constructed. These models can be used to carry out research into risk factors and safety measures designed to mitigate the risk of AVT in food processing facilities. In doing so, a page can be taken from similar research carried out in health care settings [37,38], in which pathogens, including viruses, are quantitatively measured in order to determine levels of exposure over defined distances, and safety measures are empirically tested in the setting in question [39,40]. Key questions in this body of literature include the factors that influence the survival of AVs found in particles; the distance travelled by AV containing particles; the time particles remain suspended in the air; particle size; the minimum viral load necessary to induce infection; and the link between the frequency of coughing, sneezing, and talking and the number of particles, size, and distance travelled [41,42,43].

## 3. Proposal for an AVT Model Facility

**Figure 1:**
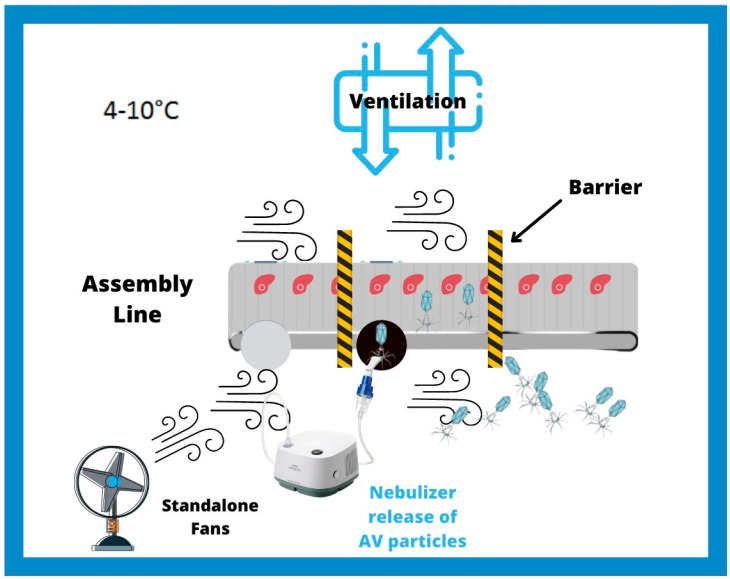
Airborne Viral Threat (AVT) laboratory model of meat processing plants. At a minimum, this model will contain an assembly line, barriers (black and yellow striped bars), a ventilation system, fans for air flow, and temperature control at 4–10 °C. An example of an experiment to carry out would be to use a nebulizer to release AV particles (blue) near the assembly line and then measure the distance the particles travel by air and determine where they settle on the surface.

**Figure 2:**
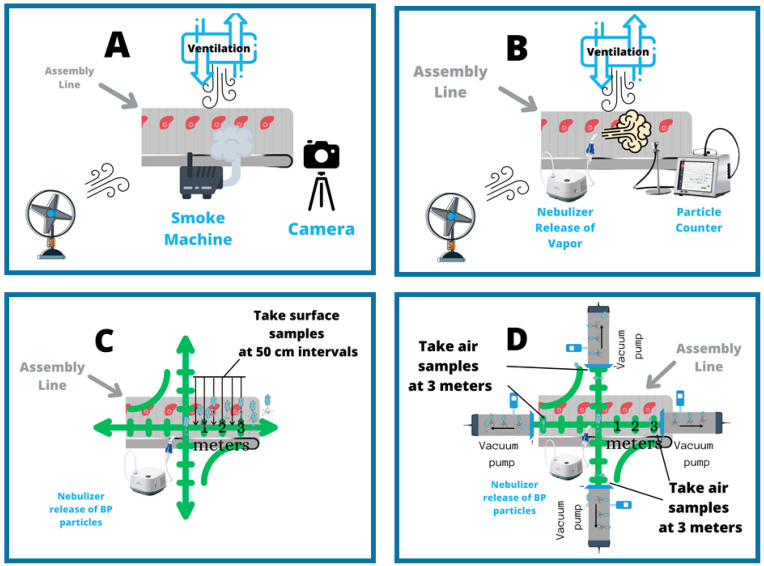
(**A**) The dynamics of air flow will be qualitatively determined by generating smoke and then documenting the movement by camera. (**B**) The dynamics of moisture particles will be quantified by releasing moisture using a nebulizer and counting particles at defined distances using a particle counter. (**C**) With each defined set up conditions, bacteriophages (BPs) (in light blue) will be released by nebuliz-er into the air in the AVT facility and the level of dispersion and distances travelled by BP viral particles will be measured over time (0 min, 10 min, 20 min, 30 min). Dispersion will be measured by (**A**) surface samples collected at each time every 50 cm for 3 meters in 4 directions (0°, 90°, 180°, 270°) from the point of BP release and then analyzed by viral plaque assay; and (**D**) air samples will be taken at 3 meters at 0°, 90°, 180°, 270° angles from the point of release and then analyzed by qPCR. Therefore, for every experiment there will be 24 surface samples and 4 air samples.

A proactive approach would require designing and building an AVT model facility that will simulate the conditions in MPPs and use this model to carry out research into risk factors and safety measures designed to mitigate AVT risk. At a minimum, this model will contain an assembly line, barriers, a ventilation system, fans for air flow, and a temperature control system set to 4–10 °C. (Figure 1). The research and educational tools created using this model will thus help to prevent the emergence of AVT in the food industry in the future. As a first step towards demonstrating that the AVT model can be reproducibly employed to measure AV dynamics, assembly line speed, barriers, ventilation conditions, and frequency of air replacement should be thoroughly studied in order to determine the effects of these factors on (1) the dynamics of air flow, (2) the dynamics of moisture particles, and (3) the dynamics of bacteriophage (BP) particles used as standins for the highly infectious SARS-COVID-19 virus (Figure 2A–D). Ultimately this model facility should mimic conditions in an MMP factory as closely as possible.

**Dynamics of Air Flow** In order to achieve an initial qualitative view of the effects of specific factors on air flow dynamics, a smoke machine should be used to create a smoke trail that can be used to track air flow in the AVT model. Photographs could be taken at discrete time points so that we will get information on the direction and time scale of the air movements (Figure 2A).

**Dynamics of Moisture Particles** In order to gain an initial model of the effects of specific factors on particle movements, the movement of moisture particles could be measured by using a nebulizer to release moisture in the AVT model and then placing a particle counter at discrete distances from the moisture release point (Figure 2B). The particle counter will count the number of moisture particles. In this way, the number of moisture particles over time can be quantified at given distances. This data will provide insight into the timescale of particle movements and an estimate of the distance at which particles begin to settle on surfaces. 

**Dynamics of BP particles** BP particles can be released using a commercial nebulizer at different locations in the AVT model (Figure 2C,D). The dispersion of the BP particles could be determined by plotting the reduction in the number of BP particles over distances travelled [44,45,46,47]. The direction and the distance traveled by the BPs can be monitored by taking both surface samples (Figure 2C) and air samples (Figure 2D). The air sampling methodology should employ vacuum filters as seen in studies found in the literature [48,49]. Air samples should be collected using vacuum sampling devices. The intake end of the device should be placed 3 m from the point of initial release in four directions (0°, 90°, 180°, 360°). This constant flow of air containing BPs will thus pass through, and virus particles will be deposited from the air onto a membrane. The number of particles can be determined by virtual plaque assays, in the case of surface samples, and by qPCR in the case of air samples.

## 4. Conclusions

The knowledge necessary to establish science-based safety measures in MPPs will be generated only by rigorously and empirically studying MPP AVT risk factors. Among the factors identified so far are work timing and organization, workflow layout and worker proximity, mask wearing, as well as temperature, humidity, and airflow as a result of climate control design. Until now, these factors have only been adjusted reactively during or after outbreaks. These factors still need to be systematically investigated in order to gain quantitative knowledge about their relative impacts on AVT safety. This knowledge can then be used to develop MPP-specific food safety procedures in order to help to prevent the transmission of AV viruses in MPPs during the AV-based pandemics that are likely to occur in the future. An AVT model will always be limited by the danger of handling SARS-COVID-19. Therefore, non-pathogenic model viruses or other type of particles need to be substituted. These models will also be limited by the ethical concerns and the necessity of creating environments that simulate the meatpacking environment without actually being a meatpacking facility containing the vulnerable workers themselves. Nevertheless, we must begin an MMP AVT model of the type proposed in order to begin the task of preparing for the inevitable future AV pandemics. Thus, it will be possible to more systematically define effective measures related to work organization, factory layout, and climatic and airflow conditions within meat processing facilities in order to dampen the spread of AVs, enhance worker and food-safety, and prevent food shortages.

It is critical to switch from a reactive to a proactive approach in facing future AV threats. The current approach is to make piecemeal and inconsistent changes to safety procedures only AFTER the effects on workers and the industry are observed. In addition, these safety measures were clearly not enough to stem the tide of new infections or to prevent facilitate shut downs. In order to protect both the health of MPP workers and the meat industry, a more systematic and proactive approach is required that is carried out well in advance of the next AV pandemic. With the resulting knowledge in hand, the effects of the next pandemic can be mitigated. The case for a comprehensive AV model of MPP facilities is clear.

## Figures and Tables

**Table 1:** COVID-19 clusters in food processing settings reported on in media as of 6 July 2020. Taken from database https://bit.ly/3ar39ky (accessed on 13 March 2021). Meat and poultry processing (MPP) facility names are shown in bold.

Installation	Country	Locality	Date Published/Accessed	Total Number of Cases Per Cluster	Final Attack Rate
**Cedar Meats Australia**	Australia	Melbourne	22 May 2020	67	
Vegetable processing plant	Canada	Oppenheimer Group, Coquitlam, B.C.	16 May 2020	3	
**Poultry **	Canada	Coquitlam	29 April 2020	50	
**Poultry plant**	Canada	United Poultry, Canada	08 May 2020	35	
**Westfleisch meat processing plant**	Germany	North Rhine-Westphalia, Coesfeld	08 May 2020	151	
**Meat processing plant**	Germany	North Rhine-Westphalia, Oer-Erkenschwick	08 May 2020	33	0.026
**Westcrown**	Germany	Dissen	21 May 2020	146	
Fish factory	Ghana	Tema	11 May 2020	534	
**Meat processing plant**	Netherlands	Vion, Groenlo, The Netherlands	22 May 2020	45	
**Meat processing plants**	USA	South Dakota	19 April 2020	518	
**Meat processing plants**	USA	Iowa	19 April 2020	84	
**Meat processing plants**	USA	Iowa	19 April 2020	177	
**Meat processing plant**	Germany	Rheda-Wiedenbrück/Gütersloh	20 June 2020	1029	0.158
**Chicken factory**	UK	2 Sisters in Llangefni, Anglesey	25 June 2020	200	0.357
Pladis biscuit factory	UK	Leicester	25 June 2020	5	
**Kober meat factory**	UK	Kirklees	25 June 2020	165	
**Kepak meat factory**	UK	Merthyr Tydfil	25 June 2020	34	
Rowan Foods meat factory	UK	Wrexham	25 June 2020	70	
Princes canned produce factory	UK	Wisbech	25 June 2020	14	0.034
Walkers crisps factory	UK	Leicester	01 July 2020	28	0.02
**Tyson Poultry Processing**	USA	Wilkes, North Carolina	20 July 2020	570	0.254

**Table 2:** Risk factors for the transmission of COVID-19 in processing contexts.

Risk Factor	Range Identified as High Risk	Sources
shift length	8–12 h	[13]
prolonged close proximity	<6 feet, >15 min	[13]
not maintaining face covering		[13]
shared work spaces		[13]
shared transportation		[19]
facility size		[26]
assembly line speeds	High	[2]
relative humidity	90–95%	[29]
airflow	recirculated and unfiltered, low exchange	[30]

## Data Availability

Not applicable.

## References

[1] Crews J. (2020). Tyson Confirms Hundreds of COVID-19 Cases at Missouri Chicken Plant. Meat + Poultry.

[2] Taylor C.A., Boulos C., Almond D. (2020). Livestock plants and COVID-19 transmission. Proc. Natl. Acad. Sci. USA.

[3] Masotti F., Cattaneo S., Stuknytė M., Pica V., De Noni I. (2021). Transmission routes, preventive measures and control strategies of SARS-CoV-2 in the food factory. Crit. Rev. Food Sci. Nutr..

[4] McCarthy R.D.S. (2020). COVID-19 Meat Plant Closures. Meat + Poultry.

[5] Leclerc Q.J., Fuller N.M., Knight L.E., Funk S., Knight G.M. (2020). What settings have been linked to SARS-CoV-2 transmission clusters?. US Natl. Libr. Med..

[6] Cheboi P.K., Siddiqui S.A., Onyando J., Kiptum C.K., Heinz V. (2021). Effect of Ploughing Techniques on Water Use and Yield of Rice in Maugo Small-Holder Irrigation Scheme, Kenya. AgriEngineering.

[7] Salins S.S., Siddiqui S.A., Reddy S.V.K., Kumar S. (2020). Parametric Analysis for Varying Packing Materials & Water Temper-atures in a Humidifier. Int. Conf. Fluid Flow Heat Mass Transf..

[8] Shoup M. Frontline Food Industry Workers Prioritized for Next Phase of COVID-19 Vaccination, 21st of December 2020. https://www.foodnavigator-usa.com/article/2020/12/21/frontline-food-industry-workers-prioritized-for-next-phase-of-covid-19-vaccination.

[9] MacDonald J.O., Kenneth K.E., Handy C.R. (2020). Consolidation in U.S. Meatpacking.

[10] Fremstad S.R.H.J., Brown H. (2020). Meatpacking Workers are a Diverse Group Who Need Better Protections Center for Economic and Policy Research. https://cepr.net/meatpacking-workers-are-a-diverse-group-who-need-better-protections/.

[11] Yao M., Zhang L., Ma J., Zhou L. (2020). On airborne transmission and control of SARS-CoV-2. Sci. Total Environ..

[12] Mayer J. (2020). How Trump is Helping Tycoons Exploit the Pandemic. https://www.newyorker.com/magazine/2020/07/20/how-trump-is-helping-tycoons-exploit-the-pandemic.

[13] Durand-Moreau Q.A.A., Mackenzie G., Bowley J., Straube S., Chan X.H., Zelyas N., Greenhalgh T. COVID-19 in Meat and Poultry Facilities: A Rapid Review and Lay Media Analysis 2020. https://www.cebm.net/covid-19/what-explains-the-high-rate-of-sars-cov-2-transmission-in-meat-and-poultry-facilities-2/.

[14] WHO (2020). The Best Time to Prevent the Next Pandemic Is Now: Countries Join Voices for Better Emergency Preparedness.

[15] Centers for Disease Control and Prevention Interim Guidance from CDC and the Occupational Safety and Health Administration (OSHA): Meat and Poultry Processing Workers and Employers. https://www.cdc.gov/coronavirus/2019-ncov/community/organizations/meat-poultry-processing-workers-employers.html.

[16] Nagdalian A.A., Rzhepakovsky I.V., Siddiqui S.A., Piskov S.I., Oboturova N.P., Timchenko L.D., Lodygin A.D., Blinov A.V., Ibrahim S.A. (2021). Analysis of the Content of Mechanically Separated Poultry Meat in Sausage Using Computing Microtomography. J. Food Compos. Anal..

[17] Pimentel D., Whitecraft M., Scott Z.R., Zhao L., Satkiewicz P., Scott T.J., Phillips J., Szimak D., Singh G., Gonzalez D.O. (2010). Will Limited Land, Water, and Energy Control Human Population Numbers in the Future?. Hum. Ecol..

[18] GPMB (2019). The World at Risk: Annual Report on Global Preparedness for Health Emergencies.

[19] Repko M., The Meat Supply Chain is Broken (2020). Here’s Why Shortages are likely to Last during the Coronavirus Pandemic. CNBC.

[20] Skerritt J., Shanker D., Hirtzer M. (2020). Meat Shortages Reopen Costly Path to Smaller U.S. Plants. Bloom. Bus..

[21] Huff A.G., Beyeler W.E., Kelley N.S., McNitt J.A. (2015). How resilient is the United States’ food system to pandemics?. J. Environ. Stud. Sci..

[22] Günther T., Czech-Sioli M., Indenbirken D., Robitaille A., Tenhaken P., Exner M., Ottinger M., Fischer N., Grundhoff A., Brinkmann M.M. (2020). SARS-CoV-2 outbreak investigation in a German meat processing plant. EMBO Mol. Med..

[23] Siddiqui S.A., Ahmad A. (2020). Implementation of Thin-Walled Approximation to Evaluate Properties of Complex Steel Sec-tions Using C++. SN Comput. Sci..

[24] Siddiqui S.A., Ahmad A. (2020). Implementation of Newton’s Algorithm Using FORTRAN. SN Comput. Sci..

[25] Swanson A.Y.-B.D. (2020). Trump Declares Meat Supply ‘Critical,’ Aiming to Reopen Plants. The New York Times.

[26] Abott C. (2020). CORONAVIRUS OUTBREAKS AT TWO CALIFORNIA CHICKEN PLANTS. Successful Farming.

[27] Baka A., Cenciarelli O., Kinross P., Penttinen P., Plachouras D., Semenza J., Suetens C., Weist K. (2020). Heating, Ventilation and Air-Conditioning Systems in the Context of COVID-19.

[28] (2004). EC (2004) Regulation (EC) No 852/2004 of the European Parliament and of the Council. Off. J. Eur. Union..

[29] Waltenburg M. (2020). Coronavirus Disease among Workers in Food Processing, Food Manufacturing, and Agriculture Workplaces Center for Disease Control-Dispatch. https://wwwnc.cdc.gov/eid/article/27/1/20-3821_article.

[30] Gibb R., Redding D.W., Chin K.Q., Donnelly C.A., Blackburn T.M., Newbold T., Jones K.E. (2020). Zoonotic host diversity increases in human-dominated ecosystems. Nature.

[31] Dyal J. (2020). COVID-19 among Workers in Meat and Poultry Processing Facilities―19 States, April 2020. Morbidity and Mortality Weekly Report (MMWR).

[32] Sampath S.S., M C.P.S., Shetty S. (2015). DETERMINATION OF POWER INHYDROELECTRIC PLANT DRIVEN BY HYDRAM: A PERPETUAL MOTION MACHINE TYPE 1. Int. J. Multidiscip. Res. Modern Educ..

[33] Suranjan Salins S., Anusha Siddiqui S., Reddy S.V.K., Kumar S. (2021). Experimental Investigation on the Performance Parameters of a Helical Coil Dehumidifier Test Rig. Energy Sources, Part A Recover. Util. Environ. Eff..

[34] Bischoff W., Russell G., Willard E., Stehle J. (2019). Impact of a novel mobile high-efficiency particulate air–ultraviolet air recirculation system on the bacterial air burden during routine care. Am. J. Infect. Control.

[35] Bischoff W.E., Swett K., Leng I., Peters T.R. (2013). Exposure to Influenza Virus Aerosols During Routine Patient Care. J. Infect. Dis..

[36] WHO (2020). Recommendation to Member States to Improve Hand Hygiene Practices Widely to Help Prevent the Transmission of the COVID-19. https://www.who.int/docs/default-source/inaugural-who-partners-forum/who-interim-recommendation-on-obligatory-hand-hygiene-against-transmission-of-covid-19.pdf.

[37] Eykelbosh A. (2020). Physical Barriers for COVID-19 Infection Prevention and Control in Commercial Settings.

[38] Han J., Zhang X., He S., Jia P. (2020). Can the coronavirus disease be transmitted from food? A review of evidence, risks, policies and knowledge gaps. Environ. Chem. Lett..

[39] KSU (2020). Grant Supports Research to Mitigate COVID-19 in Meat and Poultry Processing Facilities K-State News. https://www.k-state.edu/media/newsreleases/2020-09/covid-meat-processing91420.html.

[40] Nelson C.C., Baker M.G., Peckham T.K., Seixas N.S. (2020). Estimating the burden of United States workers exposed to infection or disease: A key factor in containing risk of COVID-19 infection. PLoS ONE.

[41] Zuber S., Brüssow H. (2020). COVID 19: Challenges for virologists in the food industry. Microb. Biotechnol..

[42] Samara F., Badran R., Dalibalta S. (2020). Are Disinfectants for the Prevention and Control of COVID-19 Safe?. Health Secur..

[43] Zhao L., Qi Y., Luzzatto-Fegiz P., Cui Y., Zhu Y. (2020). COVID-19: Effects of Environmental Conditions on the Propagation of Respiratory Droplets. Nano Lett..

[44] Inagaki H., Saito A., Sugiyama H., Okabayashi T., Fujimoto S. (2020). Rapid inactivation of SARS-CoV-2 with deep-UV LED irradiation. Emerg. Microbes Infect..

[45] Fernstrom A., Goldblatt M. (2013). Aerobiology and Its Role in the Transmission of Infectious Diseases. J. Pathog..

[46] Pottage T., Richardson C., Parks S., Walker J.T., Bennett A.M. (2007). Evaluation of hydrogen peroxide gaseous disinfection systems to decontaminate viruses. J. Hosp. Infect..

[47] WHO (2020). Considerations for Public Health and Social Measures in the Workplace in the Context of COVID-19.

[48] Siddiqui S.A., Ahmad A. Dynamic Analysis of an Observation Tower Subjected to Wind Loads Using ANSYS. Proceedings of the 2nd International Conference on Computation, Automation and Knowledge Management (ICCAKM).

[49] Siddiqui S., Sampath S.S., M C.P.S. (2016). Determination of Endurance limit and stresses in grooved mild steel. Proceedings of the 9th International Conference on Latest Trends in Engineering and Technology (ICLTET’2016).

